# Long-Term Food Variety and Dietary Patterns Are Associated with Frailty among Chinese Older Adults: A Cohort Study Based on CLHLS from 2014 to 2018

**DOI:** 10.3390/nu14204279

**Published:** 2022-10-13

**Authors:** Jiajun Zhang, Qi Wang, Wenting Hao, Dongshan Zhu

**Affiliations:** 1 Centre for Health Management and Policy Research, School of Public Health, Cheeloo College of Medicine, Shandong University, Jinan 250012, China; 2 NHC Key Lab of Health Economics and Policy Research, Shandong University, Jinan 250012, China; 3 Department of Epidemiology, School of Public Health, Cheeloo College of Medicine, Shandong University, 44 Wenhuaxi Road, Jinan 250012, China

**Keywords:** food variety, dietary patterns, frailty index, the elderly

## Abstract

(1) Objective: To examine the association between posterior-derived dietary patterns, food variety, and frailty measured by frailty index (FI) in Chinese elderly. (2) Method: A cohort study based on the Chinese Longitudinal Healthy Longevity Survey (CLHLS) from 2014 to 2018 was conducted among older adults. The food variety was defined by the food variety score (FVS), which was calculated using the frequency of food categories consumption. Dietary patterns were obtained using factor analysis. A FI composed of 38 health deficits was used to measure subjects’ frailty status. Logistic regression analyses were performed to explore the correlation between dietary factors and the incidence of frailty. (3) Results: Compared with low FVS, a high dietary diversity score at baseline was not associated with a reduced incidence of frailty after four years. Regarding long-term food variety, compared with the low variety maintained group, people with high variety maintained were associated with a lower risk of frailty (0.59, 95%CI 0.39–0.90). Adherence to the “egg-bean-pickle-sugar pattern” and “fruit-vegetable-meat-fish pattern” was associated with a lower risk of frailty. (4) Conclusion: Maintaining high food variety and adherence to two patterns, i.e., the egg-bean-pickle-sugar pattern and fruit-vegetable-meat-fish pattern, could reduce the incidence of frailty among Chinese older adults.

## 1. Introduction

According to the report from World Health Organization, the number of adults aged more than 60 years has reached a billion in 2020 worldwide and is expected to reach 1.4 billion and 2.1 billion in 2030 and 2050 [1]. In China, the elderly population aged 60 years and older has also reached 300 million in 2030 and is expected to reach 487 million in 2050 [2]. With aging, older adults tend to become vulnerable, leading to family and social burdens [3]. Frailty, an age-related clinical syndrome characterized by a decline in the function of multiple physiological systems and an increased vulnerability to acute stressors, is prevalent among older people and jeopardizes their health status [4]. It is often associated with an increased risk of adverse health outcomes, such as mortality, hospitalization, falls, disability, fractures, and high health expenditure among the elderly [5,6].

Although frailty could cause many negative health outcomes, it is proven to be changeable and reversible [7,8]. Therefore, identifying a modifiable risk factor and developing an intervention to delay, stop, or even reverse frailty is of great significance for healthy aging [9]. Recently, some interventions on specific dietary patterns, nutrient substances, and supplementation were successfully conducted among older adults, indicating nutrition is a feasibly modifiable factor for the elderly [10,11,12,13]. Meanwhile, a high-quality diet has been shown to reduce the risk of older adults’ mortality, physical functional limitations, cognitive decline, and psychological stress in many studies [14,15]. However, evidence on the associations between diet, especially food variety and dietary patterns, and frailty is limited in China.

In spite of many studies demonstrating the significant association between dietary diversity and frailty [16,17,18,19,20,21,22], most of them are cross-sectional studies. Only two studies were from China. One focused on cognitive frailty [21], and another only paid attention to fruit and vegetable variety [22]. As for dietary patterns, some researchers have argued that the Mediterranean diet, “traditional” diet, and prudent diet were associated with a lower risk of frailty among western people [23,24,25]. However, dietary preferences and cooking styles differ significantly between western and eastern populations. To date, only three studies explored food patterns in Chinese older adults [26,27,28], and only one was a longitudinal study. Thus, evidence on food patterns and frailty among Chinese older adults is limited. In addition, the majority of research on food variety, dietary patterns, and frailty uses the frailty phenotype as an endpoint. Frailty phenotype defines frailty using five physical frailty symptoms without treating frailty as holistic [29]. Compared with the frailty phenotype, frailty index (FI) takes a more comprehensive approach to indicate frailty [30], but studies using FI as an endpoint are scarce.

Therefore, to provide more insight into food variety, dietary patterns, and their associations with FI, we aim to: (1) explore the association between food variety and FI in Chinese older adults and (2) identify and examine the relationships between dietary patterns and frailty. We hypothesized that high food variety and specific dietary patterns might be associated with reduced risk of frailty.

## 2. Materials and Methods

### 2.1. Data Collection and Population

**Figure 1:**
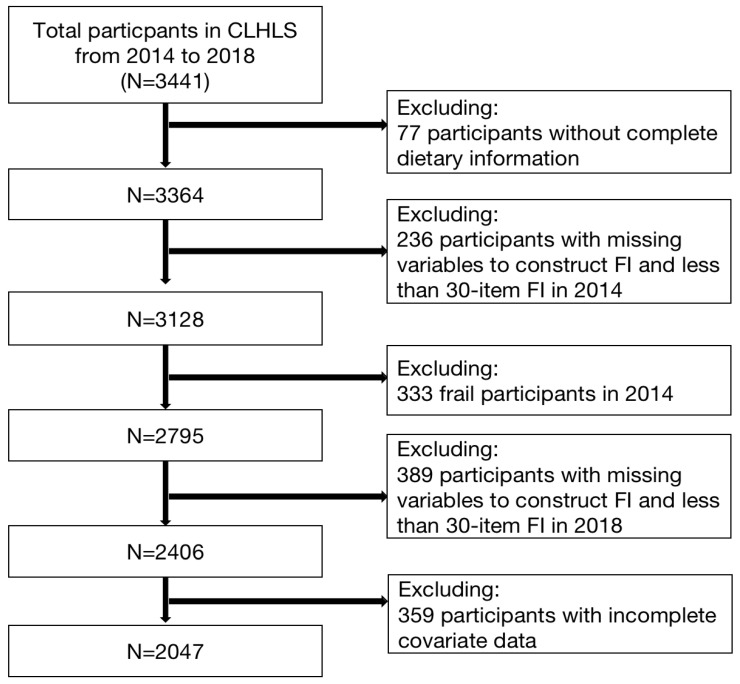
Flowchart showing the selection of the participants enrolled in the CLHLS.

Data were retrieved from the Chinese Longitudinal Healthy Longevity Survey (CLHLS) study. CLHLS is a prospective cohort study established in 1998 to explore the determinants of longevity in older adults. Samples were obtained from 22 of China’s 34 provinces, making this a nationally representative survey. A multi-stage non-equal target random sampling method was adopted, in which about 50% of counties, county-level cities, and municipal districts in all survey provinces were randomly selected as survey areas, and the areas involved were divided into “large sample counties” and “small sample counties” for the survey. Data were collected by centralized trained investigators who were assessed to be qualified for duty. Inquirers surveyed both surviving older adults and adult children of deceased older adults. The survey base period began in 1998, followed by follow-up surveys in 2000, 2002, 2005, 2008–2009, 2011–2012, 2014, and 2018–2019. The data quality of the CLHLS has been systematically evaluated [31,32]. In this study, participants who were tracked in both 2014 and 2018 were included. Finally, 2047 participants were included in this study in accordance with the inclusion and exclusion criteria in Figure 1.

### 2.2. Food Variety Measurement

The food variety score (FVS) was used to evaluate the diversity of foods consumed by older adults in 2014 and 2018. The FVS was calculated by measuring the frequency of intake of 13 foods from the CLHLS questionnaire. The 13 foods included fresh fruits, vegetables, meat, fish, eggs, soy products, salted vegetables, sugar, garlic, dairy products, nut products, mushroom plants, and tea. Frequency of fruits and vegetables intake included four optional responses: daily, often, occasionally, and hardly ever. Respondents were given a score of 1 if they answered daily or often, and 0 if they answered occasionally or hardly ever. Frequency of intake of the rest 11 foods included five options: daily, weekly, monthly, occasionally, and almost never. Respondents were given a score of 1 if they answered daily or weekly and 0 if they answered monthly or occasionally or almost never. The final FVS of each respondent was obtained by summing all food scores, ranging from 0 to 13. The higher the score, the more diverse the food intake. Based on previous studies, we defined a score less than 7 as low FVS and a score greater than or equal to 7 as high FVS [33].

Long-term food variety changes were obtained by comparing the FVS in 2014 and 2018 years. Respondents were considered having maintained high dietary diversity over time if his/her score was ≥7 in both surveys. Respondents were considered having declined dietary diversity if his/her score was ≥7 in 2014 wave and less than 7 in 2018. Respondents with a score less than 7 in the first wave and with a score ≥7 in the second wave were considered having increased dietary diversity. Respondents with a score less than 7 in both surveys were considered having maintained low dietary diversity over time.

### 2.3. Dietary Pattern Measurement

Dietary patterns in the 2014 wave were extracted using exploratory factor analysis. According to the findings of the factor analysis of each food intake, including eigenvalues, gravel maps, and the interpretability of each factor, the number of factors was established. The participant’s factor score for each pattern was then determined by calculating the intake frequency of each food and its factor loading. A higher score for a particular dietary pattern indicated a stronger preference for that dietary pattern. Four dietary patterns were identified in our study, i.e., “milk-nut-mushroom or algae pattern”, “egg-bean-pickle-sugar pattern”, “fruit-vegetable-meat-fish pattern”, and “tea pattern”. Each dietary pattern score was used to classify the participants into low- to high-quartile groups (Q1, Q2, Q3, and Q4).

### 2.4. Frailty Index

Using CLHLS data, a 38-item FI was created based on a validated and published method [34,35]. These items were chosen as they were all relevant to health status and tended to become more prevalent with age but were not fully universal in older persons. Table S1 contains information on the items and codes used to create the FI. The majority of the items had a binary option and were scored as 0 (absence) or 1 (present). To the items with ordinal options, e.g., always, often, sometimes, seldom, and rarely or never, we assigned 0, 0.25, 0.5, 0.75, and 1 to them respectively. The FI score, which ranged from 0 to 1, was calculated for each responder as the total of the item scores divided by the number of items they possessed. Participants were disqualified if they possessed fewer than 30 items. After calculating the FI score, we used the following cutoff points from a previous study to classify participants as non-frail (FI ≤ 0.25) and frail (FI > 0.25) [36].

### 2.5. Covariates

Demographic characteristics, socioeconomic status, and health-related variables were included as covariates. Gender (female or male), age (65 to 74, 75 to 84, or 85 and older), region (urban or rural), marital status (married or partnered, or others), and living condition (alone or not alone) were all demographic characteristics. Socioeconomic status included family income [quintile 1 (lowest), quintile 2, quintile 3, or quintile 4 (highest)]. Smoking status (current, former, or never), alcohol consumption status (current, former, or never), exercise status (yes or no) were all lifestyle factors. Vitamins intake was categorized as often use or not. BMI was classified into four groups: underweight (BMI < 18.5 kg/m^2^), normal weight (18.5 kg/m^2^ ≤ BMI < 24.0 kg/m^2^), overweight (24 kg/m^2^ ≤ BMI < 28.0 kg/m^2^), and obese (BMI ≥ 28.0 kg/m^2^).

### 2.6. Statistical Analysis

We used chi-square tests to compare the baseline characteristics across two food variety groups (low FVS and high FVS). Logistic regression models were constructed to estimate the effect of food variety and four dietary patterns on risk of frailty. Analyses were performed as a crude model without any adjustment, followed by a model that was additionally adjusted for gender, age, region, marital status, living condition, and family income (model 1), and a model additionally adjusted for smoking status, alcohol consumption status, exercise status, vitamins intake, and BMI (model 2). All tests were 2-sided with a significance level of *p* < 0.05. Stata, version 16.0 (StataCorp, College Station, TX, USA), was used in all statistical analyses.

## 3. Results

Table 1 showed the baseline characteristics of Chinese older adults according to food variety. There were 871 (42.55%) and 1176 (57.45%) people with low dietary diversity and high dietary diversity, respectively. The distribution of FVS differed by groups of gender, age, region, marital status, living condition, family income, alcohol consumption status, exercise status, vitamin intake, and BMI levels. Males had a higher food diversity than females (47.69% and 37.04%). Participants’ food variety decreased as their age and family income increased. People who were married or partnered tended to intake more kinds of food than those who were not. Physically active people were more likely to have a high dietary diversity than inactive people.

Table 2 shows the association between baseline food variety and the risk of frailty. Compared with low diversity, older adults with a high dietary diversity at baseline were not associated with reduced incidence of frailty in all models, with an OR (odds ratio) and 95%CI (95% confidence interval) of 0.78 (95%CI, 0.59–1.02), 0.80 (95%CI, 0.60–1.07), and 0.81 (95%CI, 0.61–1.08), respectively. Table 2 also presents the association between long-term food variety and the risk of frailty. Compared to people in the low-variety-maintained group, people in the high-variety-maintained group had lower odds of frailty (0.59, 95%CI 0.39–0.90).

The details about KMO, Bartlett’s Test, and factor loadings of the food groups were shown in Tables S2 and S3. Four dietary patterns were identified, namely “milk-nut-mushroom or algae pattern”, “egg-bean-pickle-sugar pattern”, “fruit-vegetable-meat-fish pattern”, and “tea pattern”. Table 3 presents the associations between each dietary pattern and the risk of frailty. After adjusting all covariates, participants with the top quantile of the egg-bean-pickle-sugar pattern score in 2014 had lower odds of frailty after 4 years when compared to subjects with the lowest quantile. Participants in the highest and third quantiles of the fruit-vegetable-meat-fish pattern score had a decreased odds of frailty compared to subjects in the lowest one. No association was found between milk-nut-mushroom or algae pattern, tea pattern, and risk of frailty.

## 4. Discussion

Findings from this cohort study supported the hypothesis that high food variety and specific dietary patterns were associated with reduced risk of frailty. Specifically, the elderly, with a long-term high-variety diet, and adherence to the egg-bean-pickle-sugar pattern or fruit-vegetable-meat-fish pattern were associated with lower frailty risk over time.

To our knowledge, only two studies explored the association between food variety and frailty in China. One study found that cognitive prefrailty or cognitive frailty were associated with lower food variety among 1115 older adults in Taiwan [21]. Other research showed that fruit variety, vegetable variety, and combined variety were not related to frailty among the elderly in Hongkong [22]. It was hard to compare our results with these two studies. First, previous studies assessed frailty status using frailty phenotype which was physically orientated, whereas the FI, a holistic measurement tool, was used in our study, including social and mental aspects of frailty. Second, the latter study mentioned above focused on fruit and vegetable variety but we beyond these varieties and took 13 kinds of food into consideration. Our study added new evidence to this area. We used a comprehensive FI and found no association between baseline high food variety and risk of frailty among Chinese older adults. Using a longitudinal study design, we could also examine the long-term effect of dietary diversity and have found that compared with low maintained variety, high maintained dietary diversity was associated with a lower risk of frailty. The reasons behind this may be: eating a wider variety of foods is associated with more diversified gut microbiota in older persons [37], and gut dysbiosis could cause an innate immune response and persistent low-grade inflammation [38], which can lead to a variety of age-related degenerative diseases. Additionally, the effect of dietary diversity may be small and slow, and its effect could only be seen through long-term maintenance.

Many different patterns have been explored abroad, such as the Mediterranean diet pattern, traditional diet pattern, prudent diet pattern, the Westernized pattern, and dietary approaches to stop hypertension (DASH) [23,24,25,39,40,41,42]. Due to regional and cultural traditions, dietary patterns vary by country and population [43]. As far as we know, three studies explored dietary patterns in Chinese older adults. One study that identified three dietary patterns among 2724 Chinese community-dwelling older adults, namely “vegetables-fruits”, “snacks-drinks-milk products”, and “meat-fish”, found none of them were associated with the incidence of frailty [26]. One research found that frailty was less prevalent in the elderly who had a diet high in phytonutrient-rich plant foods, tea, omega-3-rich deep-sea fish, and other protein-rich meals, including shellfish and milk [27]. Another study indicated that higher diet quality, as defined by DASH and “protein-rich”, was related to a lower prevalence of frailty among Shanghai suburban elderly [28]. Our study identified four new dietary patterns, namely “milk-nut-mushroom or algae”, “egg-bean-condiments”, “fruit-vegetable-meat-fish”, and “tea”, and found that older adults with a higher “fruit-vegetable-meat-fish” or “egg-bean-pickle-sugar” score were associated with a lower incidence of frailty after four years. Fruits and vegetables are antioxidant-containing foods and could probably prevent frailty by lowering oxidative stress and inflammation associated with aging [44]. Meat, fish, egg, and bean are all protein-rich and contain amino acids, which increase muscle synthesis [45], preventing muscle loss and atrophy among older adults [46]. The association between pickles and frailty was limited, but research indicated that pickles include probiotics, such as lactic acid bacteria, which may support gut health, boost the immune system, increase nutrient bioavailability, and reduce lactose intolerance, allergy, and the risk of certain malignancies [47]. Thus, the protective effect of self-preserved vegetables against frailty is needed to be further explored. In addition, previous studies on sugar and frailty are inconsistent with ours [48,49]. However, the different frailty assessment tool we used makes the comparison between the former study and ours impossible. More research about sugar and frailty assessed by the FI is warranted to explore.

More randomized controlled studies should be conducted to further prove the effectiveness of specific dietary patterns confirmed via observational studies. Current clinical experiments have proven the validity of the Mediterranean diet and nutrition supplements to reduce the risk of frailty [50,51], but many posteriori-defined dietary patterns are waiting for validation. In addition to validating, figuring out how to get older adults to increase adherence to anti-frailty diets is also worth exploring among older adults. Two studies have indicated that an educational and culinary-based intervention successfully enhanced adherence to the Mediterranean-style eating pattern among breast cancer survivors and a diverse sample of families [52,53]. However, similar adherence-increasing research is scarce among the elderly and this needs to be examined in the future.

Our study has several strengths. First, unlike previous studies which adopted a cross-sectional design [26] or only focused on few food types (e.g., fruit and vegetable) [27], we adopted a prospective cohort design to examine the association between multiple food types and comprehensive frailty (not only cognitive frailty). Both baseline diet variety and long-term change of diet diversity were considered. In addition, dietary patterns (e.g., egg-bean-pickle-sugar pattern) were explored and identified based on the unique eating and cooking habits of the Chinese. Second, instead of physically frail, our study used the FI to holistically assess frailty status. Third, our research found two posteriori-defined dietary patterns associated with frailty among Chinese older adults, which could provide further insight for future improvement to nutrition guidelines. Despite these contributions, we acknowledge some limitations. First, dietary information was self-reported, which may lead to some recall bias. Second, because the CLHLS only included community-dwelling older persons, our findings may not apply to institutionalized individuals. Finally, although series of possible confounding factors were considered and adjusted in our analyses, there might be some residual and unmeasured confounding in our study.

## 5. Conclusions

In this cohort study of community-dwelling older adults, instead of baseline food variety, we observed that keeping a high dietary diversity in the long term could reduce the risk of frailty among Chinese elderly after four years. Additionally, dietary patterns of “fruit-vegetable-meat-fish” and “egg-bean-pickle-sugar” were related to a lower risk of frailty. This study prospectively demonstrated the relationships between food variety, dietary pattern, and frailty. Our findings suggest that the early initiation and long-term maintenance of a diversified diet should be encouraged to reduce the risk of frailty. Moreover, future random controlled trials might be needed to test whether dietary interventions adopting the two dietary patterns mentioned above can be effective in avoiding or delaying frailty.

## Figures and Tables

**Table 1:** Baseline characteristics of Chinese older adults by the food variety score.

Characteristic	Food Variety Score < 7	Food Variety Score ≥ 7	*p*
n (%)	871 (42.55%)	1176 (57.45%)	<0.001
Gender			<0.001
Male	505 (52.31)	554 (47.69)	
Female	366 (62.96)	622 (37.04)	
Age			<0.01
≥60 and <75	300 (52.63)	270 (47.37)	
≥75 to <85	507 (57.74)	371 (42.26)	
≥85	369 (61.60)	230 (38.40)	
Marital status			<0.001
Married or partnered	571 (52.19)	532 (47.81)	
Unmarried or others	605 (63.48)	348 (36.52)	
Region of residence			<0.01
Urban community	446 (53.80)	383 (46.20)	
Rural village	730 (59.93)	488 (40.07)	
Family income			<0.001
Quintile 1 (lowest)	358 (66.54)	180 (33.46)	
Quintile 2	331 (57.77)	242 (42.23)	
Quintile 3	236 (56.87)	179 (42.13)	
Quintile 4 (highest)	251 (48.18)	270 (51.82)	
Living conditions			<0.001
Alone	294 (54.99)	149 (45.01)	
Not alone	882 (66.37)	722 (33.63)	
BMI			<0.001
Underweight	178 (67.17)	87 (32.83)	
Normal	685 (58.55)	485 (41.45)	
Overweight	238 (50.42)	234 (49.58)	
Obese	75 (53.57)	65 (46.43)	
Smoking status			0.06
Current	222 (53.62)	192 (46.38)	
Former	132 (53.88)	113 (46.12)	
Never	822 (59.22)	566 (40.78)	
Alcohol consumption status			<0.001
Current	222 (53.62)	192 (46.38)	
Former	132 (53.88)	113 (46.12)	
Never	822 (59.22)	566 (40.78)	
Exercise			<0.001
Yes	314 (47.50)	347 (52.50)	
No	862 (62.19)	524 (37.81)	
Vitamins intake			<0.001
Often	57 (32.20)	120 (67.80)	
Not often	1119 (59.84)	751 (40.16)	

**Table 2:** Logistic regression analysis between food variety factors and the incidence of frailty.

**Food Variety Score**	**Crude Model**	**Adjusted Model 1**	**Adjusted Model 2**
<7	1 (reference)	1 (reference)	1 (reference)
≥7	0.78 (0.59–1.02)	0.80 (0.60–1.07)	0.81 (0.61–1.08)
**Food Variety Change**	**Crude Model**	**Adjusted Model 1**	**Adjusted Model 2**
Maintained low variety	1 (reference)	1 (reference)	1 (reference)
Variety gets declined	0.88 (0.63–1.24)	0.93 (0.66–1.33)	0.93 (0.65–1.33)
Variety gets increased	0.77 (0.52–1.15)	0.84 (0.55–1.26)	0.83 (0.55–1.26)
Maintained high variety	0.58 (0.40–0.85)	0.60 (0.40–0.89)	0.59 (0.39–0.90)

Model 1, adjusted for gender, age, region, marital status, living condition, and family income; Model 2, model 1 + smoking status, alcohol consumption status, exercise status, vitamins intake, and BMI.

**Table 3:** Logistic regression analysis between different dietary pattern score and the incidence of frailty.

Dietary Patterns	Crude Model	Adjusted Model 1	Adjusted Model 2
Milk–nut–mushroom or algae pattern			
Q1	1 (reference)	1 (reference)	1 (reference)
Q2	0.98 (0.68–1.40)	1.05 (0.72–1.52)	1.04 (0.72–1.52)
Q3	0.75 (0.52–1.10)	0.80 (0.54–1.19)	0.80 (0.53–1.19)
Q4	0.92 (0.64–1.32)	0.96 (0.66–1.42)	0.96 (0.64–1.43)
Egg–bean–pickle–sugar pattern			
Q1	1 (reference)	1 (reference)	1 (reference)
Q2	0.90 (0.63–1.29)	0.87 (0.61–1.26)	0.87 (0.60–1.25)
Q3	0.77 (0.54–1.11)	0.79 (0.54–1.15)	0.79 (0.54–1.15)
Q4	0.64 (0.44–0.96)	0.61 (0.41–0.90)	0.60 (0.41–0.90)
Fruit–vegetable–meat–fish pattern			
Q1	1 (reference)	1 (reference)	1 (reference)
Q2	0.66 (0.46–0.94)	0.70 (0.48–1.02)	0.71 (0.49–1.03)
Q3	0.58 (0.40–0.84)	0.58 (0.39–0.85)	0.58 (0.39–0.85)
Q4	0.64 (0.45–0.92)	0.62 (0.42–0.92)	0.63 (0.43–0.93)
tea pattern			
Q1	1 (reference)	1 (reference)	1 (reference)
Q2	0.83 (0.58–1.19)	0.88 (0.60–1.27)	0.88 (0.60–1.27)
Q3	0.77 (0.54–1.11)	0.89 (0.61–1.30)	0.90 (0.61–1.31)
Q4	0.72 (0.49–1.04)	0.93 (0.62–1.37)	0.93 (0.62–1.38)

Model 1, adjusted for gender, age, region, marital status, living condition, and family income; Model 2, model 1 + smoking status, alcohol consumption status, exercise status, vitamins intake, and BMI.

## Data Availability

The data used for this study are publicly available on the CLHLS website: https://opendata.pku.edu.cn/dataverse/CHADS (accessed on 21 June 2022).

## Supplementary Materials

The following supporting information can be downloaded at: https://www.mdpi.com/article/10.3390/nu14204279/s1, Table S1: Variables Used to Construct the Frailty Index; Table S2: KMO and Bartlett’s Test; Table S3: Dietary Pattern Factor Load.
