# Supplementary material for: Long-Term Food Variety and Dietary Patterns Are Associated with Frailty among Chinese Older Adults: A Cohort Study Based on CLHLS from 2014 to 2018
Source: Nutrients. 2022 Oct 13;14(20):4279. doi: 10.3390/nu14204279 (PMC9608682; doi:10.3390/nu14204279)
Supplement: Supplementary file 1 [file nutrients-14-04279-s001.zip › nutrients-1940559-supplementary.pdf]

Table S1. Variables Used to Construct the Frailty Index

Variables		Data Type	Cut-off point
1	Self-reported health	Ordinal	V. good = 0, good = 0.25; okay= 0.5, bad = 0.75, very bad = 1
2	Feel fearful or anxious	Ordinal	Always = 1, often = 0.75, sometimes = 0.5, seldom = 0.25, rarely or never = 0
3	Feel useless with age	Ordinal	Always = 1, often = 0.75, sometimes = 0.5, seldom = 0.25, rarely or never = 0
4	Bathing	Ordinal	Without assistance = 0, one part assistance = 0.5, more than one part assistance = 1
5	Dressing	Ordinal	Without assistance = 0, one part assistance = 0.5, more than one part assistance = 1
6	Toileting	Ordinal	Without assistance = 0, one part assistance = 0.5, more than one part assistance = 1
7	Transferring	Ordinal	Without assistance = 0, one part assistance = 0.5, more than one part assistance = 1
8	Continence	Ordinal	Without assistance = 0, one part assistance = 0.5, more than one part assistance = 1
9	Feeding	Ordinal	Without assistance = 0, one part assistance = 0.5, more than one part assistance = 1
10	Visual function	Binary	Can see and distinguish = 0, can see only = 0.5, can't see = 1, blind = 1
11	Rhythm of heart	Ordinal	≥ 80bpm = 1;<80bpm = 0
12	Hand behind neck	Ordinal	Both hands = 0, left hand = 0.5, right hand = 0.5, neither hand = 1
13	Hand behind lower back	Ordinal	Both hands = 0, left hand = 0.5, right hand = 0.5, neither hand = 1
14	Able to stand up from sitting	Ordinal	Yes, without using hands = 0, Yes, using hands = 0.5, no = 1
15	Able to pick up a book from the floor	Ordinal	Yes, standing = 0, Yes, sitting = 0.5, no = 1
16	Number of times suffering from serious illness in the past two years	Binary	Yes = 1, no = 0
17	Hypertension	Binary	Yes = 1, no = 0
18	Diabetes	Binary	Yes = 1, no = 0
19	Heart disease	Binary	Yes = 1, no = 0
20	Stroke or CVD	Binary	Yes = 1, no = 0
21	Bronchitis, emphysema, pneumonia, asthma	Binary	Yes = 1, no = 0
22	Tuberculosis	Binary	Yes = 1, no = 0
23	Cancer	Binary	Yes = 1, no = 0
24	Gastric or duodenal ulcer	Binary	Yes = 1, no = 0
25	Parkinson	Binary	Yes = 1, no = 0
26	Bedsore	Binary	Yes = 1, no = 0
27	Able to hear	Binary	Yes = 0, no = 1
28	Interviewer rated health	Ordinal	Surprisingly healthy = 0, relatively healthy = 0, moderately ill = 0.5, very ill = 1

29	Look on the bright side of things	Ordinal	Always = 0, often = 0.25, sometimes = 0.5, seldom = 0.75, rarely or never = 1
30	Keep my belongings neat and clean	Ordinal	Always = 0, often = 0.25, sometimes = 0.5, seldom = 0.75, rarely or never = 1
31	Make own decisions	Ordinal	Always = 0, often = 0.25, sometimes = 0.5, seldom = 0.75, rarely or never = 1
32	Housework at present	Ordinal	Almost every day = 0, not daily, but once for a week =0.25, not weekly, but at least once for a month=0.5, not monthly, but sometimes = 0.75, never = 1
33	Able to use chopsticks to eat	Binary	Yes = 0, no = 1
34	Number of steps used to turn around a 360 degree turn without help	Binary	$\geq$ 6 steps = 1, <6 steps = 0
35	Cataract	Binary	Yes = 1, no = 0
36	Glaucoma	Binary	Yes = 1, no = 0
37	Other chronic disease	Binary	Yes = 1, no = 0
38	Prostate Tumor	Binary	Yes = 1, no = 0

Table S2. KMO and Bartlett's Test

**Kaiser-Meyer-Olkin Measure of Sampling Adequacy**		**0.745**
Bartlett's Test of Sphericity	Approx. chi-Square	3147.782
	df	78
	Sig.	<0.001

Table S3. Dietary Pattern Factor Load

Food Groups	Milk-nut-mushroom or algae pattern	Egg-bean-pickle-sugar pattern	Fruit-vegetable-meat-fish pattern	tea pattern
Fruits	.063	.109	.313	-.419
Vegetables	.128	-.175	.350	-.101
Meat	-.091	-.105	.493	.234
Fish	-.203	.220	.392	.098
Eggs	-.017	.374	.015	-.147
Food made from beans	.004	.347	-.055	-.015
Self-preserved vegetables	-.111	.323	-.172	.284
Sugar	-.103	.349	-.037	-.023
Tea	.032	-.052	.084	.623
Garlic	.212	.031	-.070	.301
Milk	.315	.089	-.084	-.305
Nut	.425	-.135	-.024	.101
Mushroom or Algae	.436	-.129	.026	.023
